# Experiences of women with physical disabilities in labor and delivery assistance

**DOI:** 10.1590/0034-7167-2023-0290

**Published:** 2024-03-11

**Authors:** Camila Fernandes da Silva Carvalho, Luciana Pedrosa Leal, Rebeca Paes Barreto Ponce de Leão Vasconcelos Amorim, Cleide Maria Pontes

**Affiliations:** I Universidade Federal de Pernambuco. Recife, Pernambuco, Brazil

**Keywords:** Labor, Obstetric, Parturition, Stress Disorders, Post-Traumatic, Health Personnel, Disabled Persons, Trabajo de Parto, Parto, Trastornos por Estrés Postraumático, Personal de Salud, Personas con Discapacidad, Trabalho de Parto, Parto, Transtornos de Estresse Pós-Traumáticos, Pessoal de Saúde, Pessoas com Deficiência

## Abstract

**Objective:**

To understand the meaning attributed by women with physical disabilities to the health care received and expected during labor and delivery.

**Methods:**

Qualitative study, based on Social Network Theory, conducted through an online workshop in April 2022, with the participation of six women with physical disabilities. Data, collected through the focus group technique, underwent thematic content analysis with the assistance of the IRaMuTeQ tool.

**Results:**

Three thematic categories emerged: Challenges experienced during pregnancy; The experience within the maternity ward; and, The importance of social networks. The assistance provided by healthcare professionals sometimes differed between what was expected and what was received by women with physical disabilities during labor and delivery.

**Final Considerations:**

Experiences were predominantly negative, resulting from inappropriate professional conduct due to ableist attitudes. Support from members of social networks is crucial for preventing stressors.

## RESUMO

**Objetivo:**

Compreender o significado atribuído por mulheres com deficiência física à assistência de saúde recebida e esperada durante o trabalho de parto e parto.

**Métodos:**

Estudo qualitativo, fundamentado na Teoria de Rede Social, realizado por meio de uma oficina online em abril de 2022, com a participação de seis mulheres com deficiência física. Os dados, coletados por meio da técnica de grupo focal, foram submetidos à análise de conteúdo temático com o auxílio da ferramenta IRaMuTeQ.

**Resultados:**

Três categorias temáticas emergiram: Problemas vivenciados na gravidez; A experiência dentro da maternidade; e A importância das redes sociais. A assistência prestada pelos profissionais de saúde, por vezes, diferiu entre o esperado e o recebido pelas mulheres com deficiência física durante o trabalho de parto e parto.

**Considerações Finais:**

As vivências foram predominantemente negativas, resultantes de condutas profissionais inadequadas devido a atitudes capacitistas. O apoio dos membros das redes sociais é fundamental para a prevenção de fatores estressores.

## RESUMEN

**Objetivo:**

Comprender el significado atribuido por mujeres con discapacidad física a la asistencia de salud recibida y esperada durante el trabajo de parto y parto.

**Métodos:**

Estudio cualitativo, fundamentado en la Teoría de Redes Sociales, llevado a cabo mediante un taller en línea en abril de 2022, con la participación de seis mujeres con discapacidad física. Los datos, recopilados mediante la técnica de grupo focal, fueron sometidos a análisis de contenido temático con la ayuda de la herramienta IRaMuTeQ.

**Resultados:**

Emergieron tres categorías temáticas: Problemas experimentados durante el embarazo; La experiencia dentro de la maternidad; y La importancia de las redes sociales. La asistencia proporcionada por los profesionales de la salud, en ocasiones, difirió entre lo esperado y lo recibido por las mujeres con discapacidad física durante el trabajo de parto y parto.

**Consideraciones finales:**

Las experiencias fueron predominantemente negativas, resultado de conductas profesionales inadecuadas debido a actitudes capacitistas. El apoyo de los miembros de las redes sociales es fundamental para la prevención de factores estresores.

## INTRODUCTION

Access to health services for people with disabilities, despite legal support, faces obstacles such as physical, attitudinal, and organizational barriers^(1)^. In addition to these, the prejudices experienced by people with disabilities make them more vulnerable, especially when it comes to the diversity of the individual in terms of gender, race/ethnicity, social class, age/generation, religion, and sexual orientation^(2)^. Therefore, women with physical disabilities experience a double vulnerability, due to both their body condition and gender, which can influence aspects of their lives, such as caring for their health.

The sexual and reproductive health of women with physical disabilities encounters persistent myths and prejudiced ideas in society. Those around them may perceive them with a childlike or asexual view, negatively impacting their sexuality, as they see themselves as incapable of having an active sexual life^(2,3)^. Healthcare professionals may also adopt discriminatory attitudes regarding their sexuality and show gaps in assistance. Such attitudes can interfere with the education and promotion of sexual and reproductive health, leading to vulnerability to sexually transmitted infections, sexual abuse, and unplanned pregnancies^(2)^.

When labor and delivery are mishandled, it can create a gateway to the development of postpartum post-traumatic stress, including the possibility of experiencing negative memories, nightmares, or uncontrollable emotions, resulting in avoidance behavior towards situations, people, or places related to trauma. Postpartum post-traumatic stress can interfere with relationships and individual health, or even the bond between mother and child^(4,5)^.

One possible alternative to reduce the occurrence of postpartum post-traumatic stress is for healthcare professionals to work with the social network of women in the perinatal cycle. Social Network Theory posits that people are in constant social interaction, which can generate support or limitations in the relationship between network members^(6)^. By understanding the dynamics of social interaction, healthcare professionals can promote health education to make the response of the social network favorable to the needs of women with physical disabilities.

The assistance of healthcare professionals, integrated into the social network of women with physical disabilities during labor and delivery, can positively or negatively influence their childbirth experience. There are reports of women with disabilities having predominantly negative experiences with healthcare professionals in maternity wards, but the repercussions of this care on their lives have been little explored^(7,8)^. Therefore, to understand the in-depth and subjective context of assistance regarding their needs and vulnerabilities, it is necessary to give a voice to women with physical disabilities about their experiences, as well as their expectations regarding the fulfillment of their demands by the healthcare team.

## OBJECTIVE

To comprehend the significance attributed by women with physical disabilities to the health care received and expected during labor and delivery.

## METHODS

### Ethical Considerations

The study was conducted in accordance with national and international ethical guidelines, receiving approval from the Research Ethics Committee of the Federal University of Pernambuco. Participants’ identities were preserved through the use of pseudonyms (names of public figures with physical disabilities), chosen by the participants themselves during the workshop, replacing their actual names.

### Theoretical-Methodological Framework

The theoretical framework was based on the constructs of Sanicola’s Social Network Theory^(6)^. A social network comprises a web of interpersonal and social relationships where individuals construct their identity and sense of belonging. When facing individual needs, they seek emotional, instrumental, or informational support or develop self-support within the social network. This network can be primary, with ties of intimacy, trust, and reciprocity, and secondary, establishing connections with institutions and organizations when demands are not met by the primary network^(6)^.

The methodological framework was guided by the stages of a workshop, a strategy providing a confrontation of realities and political and social awareness^(9)^. These stages include participant introductions with a verbal and ethical agreement, guidance, and facilitation of the discussion on the theme, and synthesis of what was discussed^(10)^.

The workshop methodology was integrated with the focus group technique for data collection. This technique allows a homogeneous group of participants to discuss a problem collaboratively, generating a new conception of the phenomenon or a more in-depth analysis. A moderator facilitated the discussion without opposing or influencing responses, and assistants recorded the gathered information^(10)^.

### Study Type

This is an exploratory, descriptive, and qualitative research, allowing for in-depth and subjective insights through participants’ narratives^(11)^. The research scope was guided by the Consolidated Criteria for Reporting Qualitative Research (COREQ)^(12)^.

### Study Setting

The research was conducted with participants residing in Recife, the capital of the state of Pernambuco, in northeastern Brazil. The workshop took place virtually, in agreement with the participants, due to the COVID-19 pandemic and the challenge of finding a physically accessible location that allowed for the recommended social distancing at the time to prevent the spread of the novel coronavirus.

### Data Source

Sample selection was intentional, considering the recommended sample size for focus groups between 6 and 12 participants^(9,10)^. Six women participated in the workshop, meeting inclusion criteria: women with physical disabilities, over 18 years old, who experienced labor and delivery in maternity hospitals in Recife/PE. Women with multiple disabilities and those requiring intensive therapy during the perinatal period were excluded.

### Data Collection and Organization

The workshop took place in April 2022 via video conference on the Google Meet® platform, lasting two and a half hours. Participant identification relied on support from Non-Governmental Organizations (NGOs) focused on people with disabilities, along with promotion on social media platforms (Instagram® and Whatsapp®). Invited women were contacted via Whatsapp to confirm eligibility criteria. The study’s objective and methodological procedures were explained, emphasizing the need for voluntary participation.

The Online Informed Consent Form (ICF) was sent the day before the workshop, and on the event day, it was read aloud to participants. Thus, verbal consent for participation in the research was obtained and recorded online from all participants. This methodological strategy was conducted by trained members of the research group “Nursing in women’s health in the context of the family,” through pre-data collection orientation meetings. The workshop’s duration was three hours, developed in three stages.

### First Stage - Welcoming and Socialization among Participants and the Research Team

Initially, each participant individually answered questions about sociodemographic data to characterize the sample (age, number of living children, type of delivery, and the child’s birthplace). Participants then chose a pseudonym from options of female public figures with physical disabilities to preserve their identity. The chosen names included Anita Malfatti (Italian-Brazilian painter), Flávia Cintra (journalist and writer), Frida Kahlo (Mexican painter), Mara Gabrilli (psychologist, publicist, and Brazilian politician), Maria da Penha (pharmacist and leader of women’s rights movements), Paola Antonini (Brazilian model), and Laís Souza (former Brazilian gymnast). The latter name was the only one not chosen by the participants.

After the selection, a dynamic activity linked the chosen personalities to the historical struggles of these figures in the context of physical disabilities, aiming to evoke representativeness, empowerment and motivation.

### Second Stage - Dynamic Activity Centered on the Theme, Guided by Social Network Theory

During this stage, women shared positive and negative feelings experienced on the day of their children’s birth, reflecting on the reasons for these feelings. The guiding question was: “Regarding the feelings experienced during the birth of your child, could you share them?” While sharing their experiences, the research team inquired about who was present or absent during moments of positive and negative feelings, identifying the primary and secondary social networks^(6)^ of the participants and the role of these networks in labor and delivery.

To facilitate the activity, the focus group moderator guided the discussion within the theme and workshop’s objective without influencing responses. Two data collection assistants recorded verbal and non-verbal information.

### Third Stage - Discussion and Final Activity

This stage provided a moment of reflection on what was reported. The moderator synthesized the discussion to validate collective understanding and addressed the reproductive rights of women with physical disabilities, existing public policies, and the importance of the social network, aiming to convey knowledge and empowerment. Recordings of all stages were saved, and transcription was independently performed by two data collection assistants. Subsequently, transcriptions were compared by another research team member to ensure information accuracy^(11)^.

### Data Analysis

Characterization data of the sample were presented by absolute frequency. Transcribed workshop data underwent thematic content analysis^(13)^. These data were organized into coded corpus and analyzed using the IRaMuTeQ software (Interface de R pour les Analyses Multidimensionnelles de Textes et de Questionnaires) version 0.7 alpha 2, employing Descending Hierarchical Classification (CHD) based on Reinert’s method^(14)^.

This method was chosen for its ability to group words by lexical proximity, forming vocabulary classes that represent significant similarity, aiming to identify the ideas conveyed by the textual corpus. The dendrogram was used to analyze the presented word grouping, and through semantic similarity, thematic categories emerged^(13)^. These were interpreted in light of the constructs of Social Network Theory^(6)^.

## RESULTS

Workshop participants were women with physical disabilities, all with limitations in the lower limbs, aged between 39 and 59 years, and had from zero to three living children. Only one interviewee gave birth in a private health institution. Three women underwent abdominal delivery, while one had a vaginal birth. Two participants experienced both types of delivery, with one having a cesarean and another an instrumental (forceps) delivery.

In the first stage, addressing the options of characters, three were correctly identified and associated with the empowerment of women with physical disabilities: Flávia Cintra, Maria da Penha, and Mara Gabrilli. Frida Kahlo was recognized as a public figure related to the feminist movement, but the participants did not know that she had a physical disability, despite her main works addressing this aspect.

**Figure 1:**
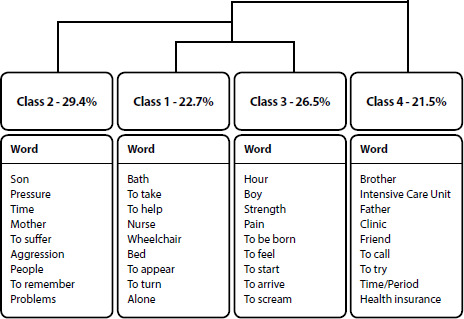
Dendrogram based on the analysis of the workshop with women with physical disabilities. Recife, Brazil, 2021

In the second stage, the workshop focused on the participants’ experiences during labor and delivery, with a focus on the assistance received and expected, stressors, and identification of the social network. Using the IRaMuTeQ program, the six transcriptions were analyzed, from which 266 text segments were constructed, totaling 9304 occurrences, with 7.17% of hapax occurrences. Of the 266 segments, 163 were classified into four classes, with 37 in Class 1, 48 in Class 2, 43 in Class 3, and 35 in Class 4. The graphical representation of the dendrogram is presented in Figure 1.

The dendrogram grouped the classes into three main groups, with the first formed only by class 2, the second group formed by classes 1 and 3, and finally, the third group formed only by class 4. From these groups, three thematic categories emerged: “Challenges experienced during pregnancy”; “The experience within the maternity ward”, and “The importance of social networks”.

### Challenges experienced during pregnancy

The first category comes from class 2, related to the obstetric problems they faced during pregnancy, such as oligohydramnios, preeclampsia, abortion, and the onset of premature labor:

*... I didn’t feel pain, only those sharp pains that I thought were him moving, but no... it was him trying to come out, that’s what the doctor told me. There was no space because the amniotic fluid dried up, and he had no more room to develop.* (Frida Khalo)

*I was at 34 weeks... placental insufficiency and losing amniotic fluid, so they admitted me. I stayed in the hospital for two weeks.* [...] *I had Pregnancy-Induced Hypertension* [PIH]*, preeclampsia, with my daughter, right?* (Anita Malfati)

*She told me to pee and said, “there’s a little cross,” I said, “what does that mean?”* [...] *she said, “it’s high blood pressure.” I had severe preeclampsia.* (Paola Antonini)

*I had him through induced labor because his little heart stopped inside my belly.* (Flávia Cintra)

Two participants experienced fetal losses in their first pregnancy, and one of them went through new episodes in subsequent pregnancies. Both reported having prenatal care in more than one institution as a way to prevent the loss of their subsequent child. These experiences negatively influenced a new pregnancy.

*I managed to stay pregnant until my eighth month; I had prenatal care* [mentioned three public maternity hospitals] *and at the health center. Even so, I couldn’t carry the pregnancy through for my baby to be born healthy.* [...] *I got pregnant again, and it was a girl. I had prenatal care in every hospital* [...] *when I was five months pregnant, I lost my daughter.* [...] *Time passed, and I thought I wouldn’t get pregnant again. I didn’t even want to because I already had the trauma of getting pregnant and losing, getting pregnant and losing, you know? I thought I wouldn’t be able to be a mother.* (Flávia Cintra)

*I had three pregnancies. Each pregnancy was different. The worst for me was the first one; I went through all the processes* [...] *when I was going to have the eldest, he passed away.* [...] *So, I had my prenatal care* [mentioned the name of the public maternity hospital] *and here at the health center. When it was time to have my second boy, when I started feeling the pain, I didn’t go to the maternity hospital. I told my sister, “I’ll only go when I can’t take it anymore because I don’t want to go through what I went through with the first one* [death during childbirth].*”* (Maria da Penha)

### The experience within the maternity ward

This category emerged from classes 1 and 3, related to the participants’ experience in the maternity ward, from their admission in labor to the immediate postpartum. Positive and negative factors were mentioned, both related to the structure of the facility and the attitudes of healthcare professionals in their care. The accessibility of maternity wards was questioned during the participants’ accounts:

*They put me in a chair because there was no stretcher. That day, the hospital had many pregnant women.* [...] *I said, “but here I can’t have my child. Because I’m sitting, I need to lie down, I need to lift my leg because I don’t have strength in that leg.” So she went and got a stretcher for me. The stretcher, besides being high, only fit my back* [laughs] *because if I turned, I would fall.* (Maria da Penha)

*The downside of the maternity ward is that it doesn’t have physical accessibility. This complicated things because, with a cesarean section, to go to the bathroom in that area where we stay to have the baby, the bathroom is not accessible. Where we stay in recovery to have the baby is also not accessible. It was a very big difficulty.* [...] *There was also no shower chair in the hospital, which made everything difficult. I had to take a shower in a regular chair.* (Flávia Cintra)

*What made it difficult for me, I believe for most wheelchair users, is the high bed* [...] *because as a wheelchair user, we need a space with accessibility. Starting from the bathrooms, after we give birth, we want to take a shower, and no one shows up to help us* [...] *I was very disappointed myself. I am already disabled, but here in my house, I don’t feel disabled. In that hospital, I was traumatized; it seemed that on that day, I really saw myself as if to say, “See? You can’t do anything.”* (Mara Gabrilli)

The labor and delivery rooms were described as hostile and traumatizing environments, with a lack of privacy for women in labor and overcrowding. Participants reported little assistance, especially those without effective contractions and, therefore, did not attract the attention of the team.

*Where the women were, everybody to give birth, everybody screaming, and I felt nothing. Imagine that horrible night!* (Frida Khalo)

*Look, it’s a room that I have never forgotten until today... I wanted to have another child, but I didn’t because of fear, because of the neglect. The room was full of women, some who were going to have a baby, others who had returned, screaming and crying, and I spent the whole night sitting in a wheelchair because there was no bed.* (Paola Antonini)

*I entered the waiting room, many people, pregnant women, many screaming, those things, and I was there calmly, sitting... they didn’t pay attention to me, I don’t know if it was because I was calmer, because I wasn’t in pain.* (Mara Gabrilli)

*There were lots of pregnant women, lots of naked women, I told my cousin that I couldn’t stand seeing so many “parakeets” of every color there.* (Maria da Penha)

Diverse feelings were reported during the process of labor and delivery, for both live and stillborn births:

*I was calm. I was calm and scared, of course, we get scared. I was calm, anxious, as we get...* (Frida Khalo)

*This childbirth, I suffered a lot. I suffered, suffered, suffered. I can’t recover, it wasn’t so fast, this loss was very difficult. Even today, we miss it when I talk about it.* (Flávia Cintra)

*The fear, the insecurity, the fear of losing the child.* [...] *So much neglect. That feeling of crying. Joy of having him, of him being well, but the sadness of neglect!* (Paola Antonini)

Participants recalled moments when they were not well-assisted by healthcare professionals during their time in the maternity ward. There were reports of poor assistance, invasive procedures without consent, and a lack of communication of bad news to laboring women:

*But when it was time to draw blood to go up, my ordeal began.* [...] *The girl* [...] *pulled the chair like this, threw me against the wall. The unfortunate pain that was in my belly, and I kept quiet.* [...] *she had a mean, brazen face; I think she was sleeping and got annoyed because she was called.* (Paola Antonini)

*I started screaming, screaming, screaming because he was crowning* [...] *I remember the nurse saying: “we’ll have to cut, so we can pull.” This time my mother was already with me and said they couldn’t cut me. I just know that I fainted, I had a spike in blood pressure, and I fainted. I don’t remember when my son was born. Then someone came, cut me, and forcibly pulled my son out.* (Anita Malfati)

*And when I had the boy, they took him away. And there I was waiting, I asked him: where’s my son? “Where’s my son? Where’s my son?” and they didn’t say he had died.* [...] *I don’t know, from the time I had him, it was almost four in the afternoon, I hadn’t taken a bath or anything, they didn’t release me to leave, and I couldn’t even leave. It was when my husband arrived that they said my boy had died.* (Maria da Penha)

*So it took a while to take me to the bathroom, which was not adapted because the two nurses had a hard time taking me out of the chair. I screamed in pain to sit in a normal chair. Then they turned on the shower* [...] *And there I was sitting, and the thing was loud when turned on. Cold water, and I taking a shower, the two nurses said: “while you take a shower there, we’ll go there and come back”... if I’m not mistaken, I stayed almost two hours there, cold, in pain, and worried because I left my little baby there by my bed* [...]. *When they came, they said: “they took another person in the wheelchair to have lunch, and there were only us, and it took a while because of the chair.”* (Mara Gabrilli)

Poor assistance was also a determining factor in causing potential future traumas, such as in a future pregnancy. The participant speaks about the need for a change in assistance for the reproductive rights of women with disabilities to be guaranteed.

*And I hope that other people with disabilities don’t go through what I went through because I wanted to have a second child and close that chapter* [...] *it never happened because the fear was so huge; I feel it as if it were today...* [...] *I hope it changes, that many disabled women can have their children, yes! That we also have the right to be mothers, be mothers as many times as God allows!* (Paola Antonini)

### The Importance of Social Networks

The third category emerged from the analysis of the results of Class 4, dealing with the influence of the social network on the birthing process of women with physical disabilities. The primary social network among the participants consisted of family members (mother, husband, cousins, sisters-in-law, brothers, and the child’s godmother) and friends who could have some influence on their health care (a nursing technician, a health plan employee, a doctor, a councilman, a clinic director).

The main positive points pointed out by the participants regarding the support from their primary social network were: promotion of support, defense against obstetric violence, assistance in their mobility, and facilitation of obstetric care.

*My sister-in-law witnessed everything and was outraged, so she informed my husband. He said, “Hold on, I’ll go there to cause a commotion, I’ll talk to the social worker because this shouldn’t happen, not at all.”* (Paola Antonini)

*My husband had to stay with me, but in the ward, we had to argue with the social worker because only he could assist me to the bathroom due to the cesarean section, which was causing too much pain.* (Flávia Cintra)

*My sister attempted to call...* [mentioned the doctor’s name]*, who was the director of a private clinic.* [...] *He said, “Look, I’m heading to the hospital* [...] *where it’s my shift. You take her, I’ll examine her, and she spends the night there.”* (Frida Khalo)

When the presence of their primary social network was denied or absent, negative repercussions occurred in the experience of pregnant women with physical disabilities, even though the women emphasized their knowledge about the right to a companion in their statements.

[...] *and I had my older sister; she was like that... I harbored a lot of resentment towards her because I expected her support and didn’t get it, right?* [...] *The nursing technician came with me the whole way, who is a childhood friend, and she stayed with me in the hospital because I was entitled to a companion.* (Frida Khalo) *But they didn’t let my husband in, I don’t know why! I was without a companion. I felt completely incapacitated. It was at that moment that I realized I couldn’t do anything, that I was worthless. Because at home, during my pregnancy, I did everything. There, I felt lost because I asked for my husband to come in, and they wouldn’t let him in to help me.* (Mara Gabrilli)

[...] *I said, “But I need a companion, CAN I call someone?” She* [the doctor] *said, “No, you can’t,” and I said, “Look, I can! Every disabled person has the right to a companion, and I won’t go in without one, no* [...]” *I know I went down. I went to the delivery area, then I called my cousin, and she came to meet me there, and that’s how it was.* (Maria da Penha)

The interaction of the pregnant condition and the disability raised concerns for their social network, either because they did not have a closer relationship or because they did not know how to deal with both existing conditions.

[...] *my husband, scared because he is also disabled, how was he going to deal with me when the time for delivery came?* [laughs]*. I went to my mother’s house* [...]. *She said, “Oh, my God! She’s going to have the boy!”. Then there was that agony, everyone not knowing what to do, the disabled person, that thing. There’s fear and care, all mixed, and you’re there in the middle not knowing anything, scared, anxious, happy to have* [the child]*, all mixed.* (Paola Antonini)

[...] *when I started releasing some liquid, my mother-in-law got worried because I am disabled, but my husband is not. So she got scared* [...]. (Maria da Penha)

The secondary social network was represented by the doctor, nursing team, general services assistant, and psychologist. Both positive and negative experiences were remarkable in labor, delivery, and immediate postpartum for the interviewees. Positive attitudes provided a sense of tranquility, especially when there was already a bond of trust before that moment. Negative experiences occurred in moments of vulnerability.

*I was calm, anxious, as one gets, but when I arrived at the hospital door, and the girl said, “she will be attended by Dr.* [the doctor’s name]*,” then I became more excited, you know? It gave me more confidence because she had treated my sister, who had a child before me, you know?* (Frida Khalo)

*And I couldn’t take it anymore; I was so weak from pain that I couldn’t even speak. A doctor arrived, looked, placed a device on my belly, didn’t even examine me, went to attend to others, forgot the device there because she had left it.* (Paola Antonini)

Specifically, regarding the nursing team, the participants mentioned that professionals, during assistance, provided support or not. When support was granted, the nursing team was considered humane. However, when help was denied, questions arose about their professional conduct toward the user, especially when they had a disability.

*On the second day, my sister-in-law, who was with me, said to the head nurse, “Can you help with giving a bath? A wheelchair for her to take a shower?” She said, “No, I can’t bring that, there’s no chair, I won’t go after it, and I have a back problem.”* (Paola Antonini)

*When I was five months pregnant, I lost my daughter* [...] *God places people to be close to us, placed a nurse who accompanied me through the whole process, didn’t let go of my hand for anything. A humane person, not just a professional.* (Flávia Cintra)

Prejudiced attitudes of healthcare professionals during assistance related to the reproductive health of women with physical disabilities were reported by the participants. This becomes evident when the doctor asks the participant’s brother for permission for tubal ligation or when the nursing team comments on the fact that a woman with physical disabilities is pregnant:

*And then she* [the doctor] *said, “We’re going to ligate because you’re already 40 years old, it’s a high-risk pregnancy. You’re not in a condition to have another one”, I said, “Okay, you can go ahead and do it.” Then she went outside to ask my brother because there’s also this story, a person with a disability... right? She went outside to ask my brother, and my brother said, “Okay!”* (Frida Khalo)

*... there was a nurse who was so rude* [...] *because they were talking among themselves like, “I don’t know why, besides her already having this problem, she decides to get pregnant. What happens is that women can’t even see underwear; they get pregnant.” Man, she doesn’t even know! I, thank God, at the time, was happily married, and she started judging me poorly, thinking that I got pregnant just for the sake of it. She didn’t know me, and they started criticizing me without even knowing me.* (Mara Gabrilli)

## DISCUSSION

The workshop participants shared experiences of healthcare assistance that, at times, deviated from their expectations during labor and childbirth. The anticipation of having their needs met was not consistently fulfilled due to ableist attitudes and prejudiced remarks that heightened social barriers.

Healthcare professionals form a secondary social network with technical competence to address the needs of parturients in a multiprofessional context. This helps in reducing post-traumatic stress in the postpartum period, vulnerability, and the risk of trauma, contributing to a healthy maternal-fetal outcome for both^(15)^. However, when adopting a restraining behavior, professionals fail to meet women’s needs, leading to the inefficiency of the secondary social network. In the care of women with physical disabilities, restraint occurs when there are prejudiced attitudes, communication failures, a lack of understanding of the client’s needs, limited professional knowledge, and a lack of integration of the woman into the care plan^(7,16,17)^.

Nursing staff assistance was frequently mentioned by participants, underscoring the significant role these secondary social network professionals play in caring for parturients with physical disabilities. Through their work, nurses can address the needs of the parturient and advocate for reproductive rights, autonomy, and the empowerment of women with physical disabilities^(18)^. However, nurses need to acquire specific knowledge about this target audience. It is emphasized that the absence of adequate nursing care can increase the vulnerability of the parturient and expose them to stressors that may lead to postpartum posttraumatic stress.

The primary social network was effective in reducing stress factors and mitigating the impact of the lack of accessibility during labor, childbirth, and immediate postpartum. Healthcare professionals can act as a bridge between primary and secondary social networks by establishing a bond with women with physical disabilities and their networks, understanding the relational dynamics among these network actors, and mobilizing appropriate support^(19)^. However, it should be noted that support cannot be imposed but offered, not limiting autonomy in decisions about one’s care^(18)^. This strengthening of bonds provides security for members of social networks in managing and meeting the needs of pregnant women with physical disabilities.

Participants’ accounts of health problems experienced during pregnancy contribute to an increased risk of perinatal complications in women with disabilities, including a high rate of cesarean deliveries among those with physical disabilities. The absence of reproductive planning and high-quality perinatal care specific to their demands and accessible are contributing factors to the increased risk of these obstetric complications^(20)^. Comorbidities and obstetric problems are understood as potential stressors for the development of postpartum post-traumatic stress due to risks to maternal-fetal health.

Participants highlighted factors that generated negative feelings, influencing their childbirth process in maternity hospitals. Among these factors are a lack of accessibility, failures in effective communication, absence or denial of a companion in maternity care, non-consensual health procedures, and negative attitudes from healthcare professionals. These social barriers impact the quality of care received from prenatal to maternity^(7,8,16,21)^.

Accessibility goes beyond architectural and urban structures, encompassing any barrier that interferes with the full experience of women with disabilities during childbirth, with autonomy and independence. Therefore, the difficulties presented by healthcare services in meeting their needs result in a lack of respect for their identity, privacy, and dignity^(16,21)^. This is evidenced in the words of one participant who did not feel limited in her home but perceived dependence due to her disability because of the lack of accessibility in the maternity ward.

Issues regarding the care received in maternity hospitals, emphasized by parturients with physical disabilities, participants in the study, should be replaced by humanitarian and respectful healthcare actions tailored to the specific needs of women with disabilities. It is emphasized that negative experiences and feelings stem from stressors generated by inhumane care, with consequent fear of childbirth or future pregnancies. The fear of childbirth can be a psychopathological condition leading to other mental health problems, such as post-traumatic stress disorder^(22)^.

The combination of physical structure with accessibility, provision of scientific knowledge, and an interdisciplinary team guides the safe work of healthcare professionals and helps build women’s confidence in the care received. For this, continuing education, access to guides and manuals on the topic, and other knowledge sources can contribute to the development of healthcare professionals’ skills in providing sexual and reproductive health care to women with physical disabilities^(21,23)^.

Empowering these women regarding their sexual and reproductive rights also enables the reduction of stressors. The participants in this study showed awareness of their rights, reducing the impact of their vulnerability and the prejudices faced during maternity. In this context, it is noteworthy that when healthcare professionals are trained to care for this population, they gain a strategic position in promoting health education to both these women and their primary social network^(24)^. These educational actions are carried out through active listening, aiming to understand the strengths, weaknesses, and needs of these women to collaboratively plan their care. When done collaboratively, it makes women with disabilities more conscious of their bodies and motherhood.

### Limitations of the Study

This research has significant limitations to consider. The first one relates to the virtual format adopted for the workshop, making it challenging to fully observe participants’ facial expressions and resulting in occasional interruptions due to internet connection failures. Both factors may have affected the capture of subjectivity in this research. Additionally, the study did not include the participation of women with other physical disabilities, such as amputations or dwarfism. Their inclusion could have offered an additional and enriching perspective, addressing specific issues related to their particular physical conditions.

### Contributions to Nursing and Health

This study provides healthcare professionals with essential insights for reflection and action focused on the needs of women with physical disabilities in the perinatal cycle. By highlighting the meaning attributed by these women to the healthcare received and expected during labor and childbirth, it aims to offer insights into maintaining high standards of quality in their care and encourage professionals to identify and reduce potential stressors.

## FINAL CONSIDERATIONS

Women with physical disabilities expect humanized healthcare that reduces stressors in maternity, allowing them to feel more secure and confident during labor and childbirth. However, not all of them experienced this care due to prejudiced attitudes, lack of accessibility, and inhumane actions present in different maternity wards, regardless of whether they are public or private institutions. However, when healthcare teams provided positive experiences, the obstetric outcome was linked to the satisfaction of women with physical disabilities.

The theme of the health of people with disabilities needs reflection, contextualized in the training of healthcare professionals, and revisited in continuing education to provide knowledge and security for healthcare meeting the needs and expectations of women with physical disabilities in their childbirth process. Specifically for nurses, whose work is centered on caring, acting in the prevention or reduction of postpartum post-traumatic stress, promoting sexual and reproductive rights, and strengthening the social networks of this population. Nurses need to discard socially conceived prejudices and seek to appropriate tools and knowledge to ensure the respect, autonomy, and accessibility of these women.

It is suggested to expand the discussion with new studies considering other disabilities and advancing to different geographical areas in Brazil to verify if there are differences in sociocultural contexts that may contribute to or interfere with healthcare in a multicultural and continental country.
